# Utilization of Hematopoietic Stem Cell Transplantation for Acute Myeloid Leukemia and Related Hospital Outcomes: A Cross-Sectional Study of US Hospitals

**DOI:** 10.7759/cureus.32821

**Published:** 2022-12-22

**Authors:** Sravani Kommuru, Sowmya Sagireddy, Adit M Patel, Hruday Raj Thoutam, Saaniya Yasmeen, Amr A Jarrad, Gagan Kaur, Viralkumar Patel

**Affiliations:** 1 Internal Medicine, Dr.Pinnamaneni Siddhartha Institute of Medical Science and Research Foundation, Vijayawada, IND; 2 Internal Medicine, Coney Island Hospital, New York, USA; 3 Internal Medicine, Byramjee Jeejeebhoy Medical College, Ahmedabad, IND; 4 Internal Medicine, Kakatiya Medical College, Warangal, IND; 5 Internal Medicine, Deccan College of Medical Sciences, Hyderabad, IND; 6 Internal Medicine, University of Jordan, Amman, JOR; 7 Internal Medicine, Sri Guru Ram Das Institute of Medical Sciences and Research, Amritsar, IND; 8 Internal Medicine, Sarasota Memorial Hospital, Sarasota, USA

**Keywords:** health services utilization, nationwide inpatient sample (nis), in-hospital outcome, hsct, hematopoietic stem cell transplantation (hsct), acute myeloid leukemia (aml)

## Abstract

Background

In this study, we aimed to provide a descriptive overview of the utilization of hematopoietic stem cell transplantation (HSCT) for the treatment of acute myeloid leukemia (AML), determine the rates of HSCT use stratified by patients’ demographic characteristics, and measure the hospitalization outcomes.

Methodology

We conducted a cross-sectional study using the Nationwide Inpatient Sample (NIS) obtained from hospitals in the United States. Our sample included 21,385 adult patients (aged ≥18 years) with a primary discharge diagnosis of AML. The sample was further grouped by inpatients who were managed with HSCT and chemotherapy as the primary procedure. We compared the demographic characteristics and hospital outcomes in AML inpatients across treatment cohorts by performing descriptive statistics and Pearson’s chi-square test. Next, we measured the differences in continuous variables (length of stay and cost) using the analysis of variance (ANOVA). All analyses were conducted using SPSS version 26 (IBM Corp., Armonk, NY, USA).

Results

The hospital-based utilization rate of HSCT was 0.4% in AML inpatients. The utilization rate of HSCT was higher in females (0.5%), African Americans (0.6%), those with median household incomes above the 50th percentile (0.5%), and those covered by private insurance (0.8%). A significantly higher proportion of AML inpatients with HSCT had depression (22.2% vs. 11.4% in total). AML inpatients receiving HSCT had significantly longer hospitalization stays and higher treatment costs than those receiving chemotherapy. The all-cause inpatient mortality was 11.6% in AML inpatients. Statistically, there were no significant differences by treatment.

Conclusions

HSCT appears to be underutilized for the treatment of AML. This treatment had a higher utilization rate in females and those from high-income families and was covered by private insurance. The utilization of chemotherapy and HSCT did not significantly differ in the presence of comorbidities, except for depression and hypertension having a higher utilization of HSCT.

## Introduction

Acute myeloid leukemia (AML) is a cancer of the blood stem cells in the bone marrow, most prominently affecting myeloid cells [1]. Among all leukemias, AML accounts for 80% of cases in the adult population. It is mainly caused by ineffective erythropoiesis and clonal expansion of immature blast cells leading to failure of the bone marrow. The incidence of AML in 2021 was reported to be 4.2 per 100,000 among women and men, and a total of 20,000 cases per year in the United States [2]. Males are at a higher risk than women with a ratio of 5:3, and more cases are seen in adults above the age of 65 compared to the younger population. The lifetime risk of men and women developing AML has been reported to be 0.5%. The mortality rate of AML has been reported to be 2.4 per 100,000 per year, with a five-year survival rate of 30.5% [3].

The standard therapy for AML involves the induction of chemotherapy with a combination of cytarabine and anthracycline [4]. The patient’s ability to tolerate the treatment and the likelihood of a cure determine if chemotherapy alone is sufficient or if an allogeneic stem cell transplant is required [4]. AML in patients over 60 years of age carries a poor prognosis and seldom resolves with chemotherapy alone. Hematopoietic stem cell transplantation (HSCT) may lead to longer survival rates in older patients with AML. Currently, 22% of HSCT receivers are older than 60 years [5]. Patients with a high risk (70-90% chance) of relapse are offered HSCT as the survival rates increase to 40-50% from 10-30% [6].

The burden of AML is not only seen in clinical settings but also financially. Across all treatment episodes, the average cost has been estimated to be $439,104 for relapsed and refractory, followed by HSCT at $329,621, high-intensity induction chemotherapy at $198,657, high-intensity consolidation chemotherapy at $73,428, and low-intensity chemotherapy at $53,082. Inpatient hospitalization is the largest contributor to cost, accounting for roughly 70% of the cost across all treatment groups [7]. The utilization of HSCT improves the quality of life within one-year post allogenic transplant when most patients return to their baseline. This highlights the economic burden and utilization of resources for the treatment of AML in the US healthcare system. A significant improvement in overall health was seen in recipients including an improvement in symptom profile but the greatest impact was seen in physical function which continued even after the first year [8]. The healthcare burden of AML is evident through the utilization of resources and direct health care, especially in cases that have relapsed or are refractory.

In this study, we provide a descriptive overview of the utilization of HSCT for the treatment of AML and determine the rates of HSCT use stratified by patients’ demographic characteristics using a Nationwide Inpatient Sample (NIS). Our second goal is to measure the hospitalization outcomes, including length of stay (LOS), cost, the severity of illness, and inpatient mortality in inpatients managed with HSCT.

## Materials and methods

Study sample

We conducted a cross-sectional study using the NIS. The NIS is hospital-based administrative data obtained from non-federal hospitals across 48 states and the District of Columbia in the United States. As the NIS is de-identified data, we were not required to take institutional review board approval for our study [9].

We included 21,385 adult patients (aged ≥18 years) with a primary discharge diagnosis of AML. The sample was further grouped by inpatients who were managed with HSCT and chemotherapy as the primary procedure.

Variables

Demographic variables including age, sex, race, primary payer, and median household income were collected. The comorbidities noted in the patient records included alcohol abuse, arthropathies, metastatic cancer, depression, diabetes with complications, hypertension, chronic pulmonary disease, obesity, and hypothyroidism. Hospital outcomes drawn from the NIS included the LOS and cost during hospitalization for the treatment of AML, the severity of illness which was measured using the all-patient refined drugs (APR-DRGs), and in-hospital mortality (all-cause) [9].

Statistical analysis

We compared the distributions of demographic characteristics and hospital outcomes in AML inpatients across treatment cohorts by performing descriptive statistics and Pearson’s chi-square test. Next, we measured the differences in continuous variables, i.e., age, LOS, and cost using the analysis of variance (ANOVA). All analyses were conducted using SPSS version 26.0 (IBM Corp., Armonk, NY, USA). Statistical significance was set at a two-sided p-value <0.05.

## Results

The hospital-based utilization rate of HSCT was 0.4% (90 out of 21,385) in AML inpatients. The inpatients in the HSCT cohort were younger compared to chemotherapy and total AML inpatients (mean age: 51.7 vs. 54.6 vs. 62.7 years). Females accounted for the majority of HSCT recipients (55.6%), and the utilization rate of HSCT was also higher in females (0.5%) compared to males (0.3%) when males formed 55.8% of total AML inpatients. Although Whites (72.2%) accounted for the majority of HSCT recipients, the rate of utilization was the highest in African Americans (0.6%), with Whites having a utilization rate of 0.4%.

The inpatients were closely distributed by median household income but the HSCT utilization was higher in those with a median household income above the 50th percentile (64.7%) for a utilization rate of 0.5%. Private insurance was the dominant primary payer to cover the HSCT inpatients (66.7%) with a utilization rate of 0.8%, which was much higher than that seen in total AML inpatients who were majorly covered by Medicare (48.7%).

A significantly higher proportion of AML inpatients with HSCT had depression (22.2% vs. 11.4% in total) and hypertension (16.7% vs. 18.6% in total), whereas there existed a statistically non-significant difference across other comorbidities by treatment, as shown in Table 1.

**Table 1:** Utilization of HSCT in the study population. *: data values with N below 10 that cannot be reported as per the data policy. SD = standard deviation; HSCT = hemopoietic stem cell transplantation

Variable	Total	Chemotherapy	HSCT	Utilization rate of HSCT in %	P-value
Number of inpatients	21385	470	90	0.4	-
Mean age (SD)	62.7 (16.7)	54.6 (16.9)	51.7 (16.9)	-	<0.001
Sex, in %					
Male	55.8	59.6	44.4	0.3	0.025
Female	44.2	40.4	55.6	0.5	
Race/ethnicity, in %					
White	71.3	64.8	72.2	0.4	<0.001
African American	11.1	15.4	16.7	0.6	
Hispanic	9.4	9.9	0	0	
Other	8.2	9.9	*	*	
Median household income, in %					
Below 50^th^ percentile	46.9	52.1	35.3	0.3	0.007
Above 50^th^ percentile	53.1	47.9	64.7	0.5	
Primary payer, in %					
Medicare	48.7	41.5	27.8	0.2	<0.001
Medicaid	10.8	12.8	*	*	
Private	34.4	37.2	66.7	0.8	
Uninsured	6.2	8.5	0	0	
Comorbidities, in %					
Alcohol abuse	1.9	1.1	0	0	0.177
Arthropathies	3.7	5.3	*	*	0.106
Metastatic cancer	1.7	*	0	0	0.341
Depression	11.4	13.8	22.2	0.8	0.001
Diabetes with complication	12.2	9.6	*	*	0.031
Hypertension	18.6	12.8	16.7	0.4	0.004
Chronic pulmonary disease	14.4	13.8	*	*	0.061
Obesity	13.7	10.6	*	*	0.110
Hypothyroidism	13.1	13.8	*	*	0.771

AML patients who received HSCT had significantly longer LOS and higher treatment costs compared to those who received chemotherapy and the total inpatients. When 73.2% of total AML inpatients had major severity of illness, about four-fifths were HSCT recipients, with a utilization rate of 0.5%. The all-cause inpatient mortality was 11.6% in AML inpatients, and, statistically, there existed no significant difference by treatment, as shown in Table 2.

**Table 2:** Hospital outcomes in acute myeloid leukemia inpatients by the procedure. *: data values with N below 10 that cannot be reported as per the data policy. SD = standard deviation; LOS = length of stay; HSCT = hemopoietic stem cell transplantation

Variable	Total	Chemotherapy	HSCT	Utilization rate of HCT in %	P-value
Mean LOS, days (SD)	18.8 (18.5)	31.7 (22.5)	52.1 (33.4)	-	<0.001
Number of days from admission to procedure	-	6.4 (8.8)	7.2 (2.5)	-	<0.001
Mean cost, $ (SD)	278,289 (374,554)	478,852 (561,789)	930,712 (667,499)	-	<0.001
Severity of illness, in %					
Minor	2.8	0	0	0	<0.001
Moderate	24.0	10.6	16.7	0.3	
Major	73.2	89.4	83.3	0.5	
Inpatient death, in %	11.6	11.7	*	*	0.986

## Discussion

The patients who achieved complete remission on treatment had much better survival rates than those who did not [10]. Along similar lines, Loke et al. reported that the risk of relapse in patients with complete remission after intensive chemotherapy was reduced by nearly 60% [11]. Allogenic HSCT is the most potent anti-leukemic therapy mediated by the graft versus leukemia effect in AML patients and is routinely used in patients with intermediate and poor risk disease [6,12]. Early detection of cases with a higher risk of relapse enables more patients with AML to receive HSCT. Our cross-sectional study on AML inpatients revealed that the overall rate of utilization of HSCT was 0.4%, with relatively higher rates in young patients, females (0.5%), African Americans (0.6%), and patients with a median household income above the 50th percentile (0.5%).

According to an evidence-based review from the American Society of Transplantation and Cellular Therapy, the cytogenic risk is the most important parameter when considering suitable candidates for allogeneic HSCT. Moreover, allogeneic HSCT in patients with complete remission is best suitable for adverse or intermediate-risk cytogenetics but not for favorable-risk cytogenetics. The results from their meta-analysis stated that allogeneic HSCT recipients showed significantly better relapse-free survival than those whose cytogenetics were adverse risk (hazard ratio (HR) = 0.69; 95% confidence interval (CI) = 0.57-0.84) or intermediate risk (HR = 0.76; 95% CI = 0.68-0.85) rather than favorable risk (HR = 1.06; 95% CI = 0.80-1.42) [13]. While considering candidates for HSCT, it is crucial to consider the potential side effects of the treatment. The most reported side effect of HSCT is severe graft-versus-host disease (GVHD) [14]. The risk of GVHD increases when a human leukocyte antigen (HLA)-matched sibling donor is unavailable for transplantation. Although the risk of GVHD is lower with autologous HSCT, there is an increased risk of relapse owing to the lack of graft versus leukemia effect and possible contamination of the graft by leukemia cells [14].

Existing literature on AML does not speak much about the healthcare and financial burden. Our cross-sectional study identified that the average LOS for patients with AML was significantly higher among patients receiving HSCT. Along similar lines, a study by Pandya et al. stated that the duration of each episode of care was the longest in patients with refractory or relapsed AML (14.77 months), followed by patients with HSCT (6.35 months). Another important finding from our study was that most people who utilized HSCT were covered by private insurance, and medicare covered only 0.2% of the patients. This finding is in line with the findings of the study by Pandya et al., which stated that the total mean episode costs were the highest in refractory or relapsed episodes ($439,104, followed by HSCT at $329,621) [7].

AML costs are significantly driven by hospitalization and medical costs, the cost of stem cell transplants, and the cost of medications. With AML treatment moving to the outpatient setting, hospitalization and medical costs are likely to decline. As more patients become eligible for improved transplant procedures, the cost of stem cell transplantation may increase, although the overall cost may decrease if new procedures lead to reduced hospital stay in patients [15]. Overall, 66.7% of HSCT patients were paid for by private insurance. The utilization rate for private insurance was 0.8%, significantly higher than Medicare coverage for total AML patients (48.7%).

There was a statistically significant difference in the percentage of AML patients with depression undergoing HSCT compared with those not receiving HSCT (22.2% in comparison to 11.4%) in total oncological conditions that often coexist with depression. Patients receiving allogeneic HSCT are at risk of poor treatment survival outcomes in addition to depression, which affects the quality of life. Consequently, most stem cell transplantation centers require pre-transplant psychiatric evaluations for patients. Depression has been identified as a major risk factor for post-transplant mortality by numerous prognostic scoring systems. A highly useful prognostic tool in allogeneic HSCT is the hematopoietic cell transplantation-specific comorbidity index [16]. Due to the severity of the conditioning regimen and the risk of GVHD, which may lead to mortality, patients are required to undergo a lengthy hospital stay that includes isolation for at least four weeks, as well as extended recovery periods. Relapse of cancer occurring after several failed treatments is not uncommon among these patients [17]. These may contribute to the high prevalence of depression in our study sample.

Our study should be viewed with some limitations in mind. To conduct our study, we used a data sample from the NIS, which does not contain individual patient-level information. It is important to note that the participants in our study were selected from an inpatient sample, so their comorbidities may differ from those in the general population. We could not include certain comorbidities, including arrhythmia, coronary artery disease, stroke, inflammatory bowel disease, and liver disease, which may impact the utilization of HSCT. There is also the possibility that the results of our study might not represent people from other races or ethnicities because our study population largely consists of Whites. Lastly, due to the nature of the NIS data, we did not have further information if LOS could impact the utilization of chemotherapy versus HSCT and post-HSCT complications, including GVHD. One of the strengths of the study is that the NIS has the capacity to build a population-based inpatient representation of associations between diseases and comorbidities. The chances of recall bias are minimal given that the NIS has primary and secondary diagnostic codes and other clinical information obtained at the time of hospitalization. Another strength of this study is its large sample size of 21,385 inpatients and data reliability as the information was coded independently of the individual practitioner, minimizing reporting bias. Moreover, the large sample size increased the power to detect differences.

## Conclusions

HSCT seems to be underutilized for the treatment of AML. This treatment was used in comparatively younger inpatients with a higher utilization rate in females and those from high-income families and covered by private insurance. The utilization of chemotherapy and HSCT did not significantly differ by the presence of comorbidities except for depression and hypertension as there was a higher utilization rate of HSCT in at-risk inpatients. AML patients who received HSCT had significantly longer hospitalization stays and higher treatment costs compared to those who received chemotherapy. The all-cause inpatient mortality was 11.6% in AML, but the utilization of chemotherapy versus HSCT did not have a statistically significant difference in mortality rates. Future studies may examine the potential factors, including GVHD and post-HSCT complications, that may affect the hospitalization stay. Moreover, it is important to understand and compare the long-term survival rate in inpatients undergoing HSCT versus chemotherapy.
